# Problematic Airway and Anesthetic Dilemmas for Achondroplastic Dwarfism in the Acute Care Setting: A Case Report

**DOI:** 10.7759/cureus.25336

**Published:** 2022-05-25

**Authors:** Son H Dang, Ara Samra, Bansi V Patel, Sebastian Sanchez-Luege

**Affiliations:** 1 Anesthesiology, Jackson South Medical Center - University of Miami Miller School of Medicine, Miami, USA

**Keywords:** anesthetic management, general anesthesia, anesthetic considerations, airway difficulties, dwarfism, achondroplasia

## Abstract

Patients with achondroplasia often present with anatomical abnormalities and altered cardiopulmonary physiology that significantly increase their perioperative risk for cardiovascular and respiratory complications (e.g., worsening ventilation-perfusion mismatch, imminent desaturation, difficult airway). We describe a 34-year-old achondroplastic male presenting with altered mentation following a traumatic subdural hematoma that necessitated emergent craniotomy evacuation. Initial attempt at intubation was complicated by rapid desaturation and bradyarrhythmia. Subsequently, the patient went into cardiac arrest requiring cardiopulmonary resuscitation. A laryngeal mask airway (LMA) was secured and fiberoptic intubation was achieved in succession. Following return of spontaneous circulation (ROSC), a repeat CT scan showed the subdural hematoma to be stable in size and neurosurgery opted to delay his surgery for conservative management and close monitoring. This case highlights the unique airway challenges and anesthetic considerations in management of achondroplastic patients.

## Introduction

Achondroplasia, also known as *Chondrodystrophia fetalis*, is the most common type of skeletal dysplasia that affects approximately 1 in 15,000 to 30,000 live births [1]. This genetic disease is caused by a gain-of-function mutation in the fibroblast growth factor receptor 3 gene on chromosome 4 that leads to defective endochondral ossification. Although inherited in an autosomal dominant pattern, about 80% of cases are found to be from new sporadic mutations [2]. Due to their erratic bone formation, these disproportionately dwarfed patients often present with other anatomical defects and altered physiological functions, and are associated with comorbidities (e.g., pectus excavatum, obstructive and restrictive lung diseases) that can severely hinder their anesthetic care. The most common anesthetic obstacles in achondroplasia primarily manifest as difficult airway and spinal features (e.g., maxillary hypoplasia, tracheobronchomalacia, foramen magnum stenosis, kyphoscoliosis, spinal canal stenosis). To avoid detrimental events such as atlantoaxial dislocation, traumatic spinal cord injury, or high spinal anesthesia from occurring, special considerations are warranted when planning the anesthetic method of choice for these patients. Despite meticulous preparation in past, there have been prior reported cases with aborted surgeries, disastrous sequalae, and even death. Our case report intricately discusses the airway challenges and highlight unique anesthetic considerations in an achondroplastic patient undergoing emergency craniotomy evacuation for a traumatically acute subdural hematoma.

## Case presentation

**Figure 1:**
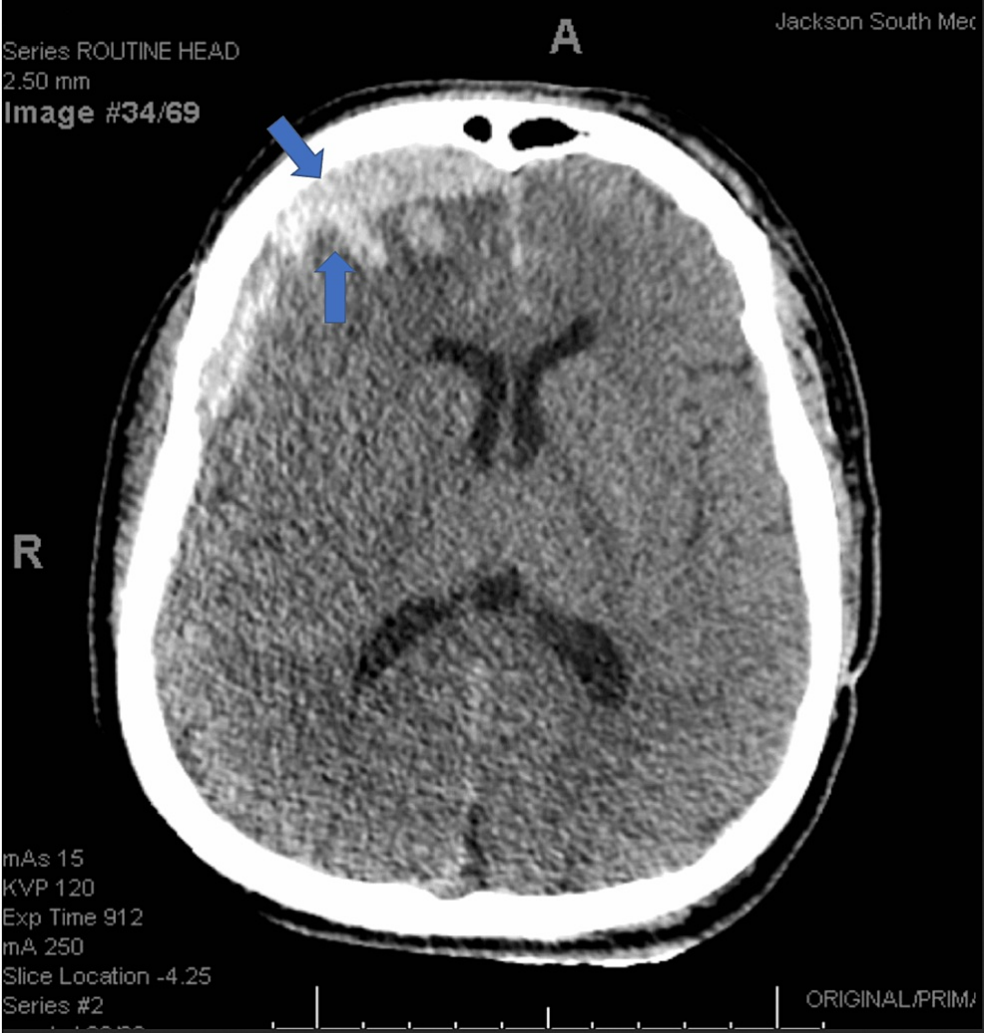
Subdural hematoma on CT of the head CT of the head demonstrates an acute 10 mm thick subdural hematoma located in the vicinity of the right frontal lobe (shown using blue arrows). There is also a 2 mm midline shift from the septum pellucidum.

A 34-year-old male with achondroplasia was brought in following an assault that resulted in a traumatic head injury. Upon arrival, the primary survey revealed a hemodynamically stable but confused male with a Glasgow coma score of 11, dwarfism features, and right-sided head swelling. The secondary survey was significant for discovering an acute 10 mm thick subdural hematoma located near the right frontal lobe, as seen in Figure 1.

**Figure 2:**
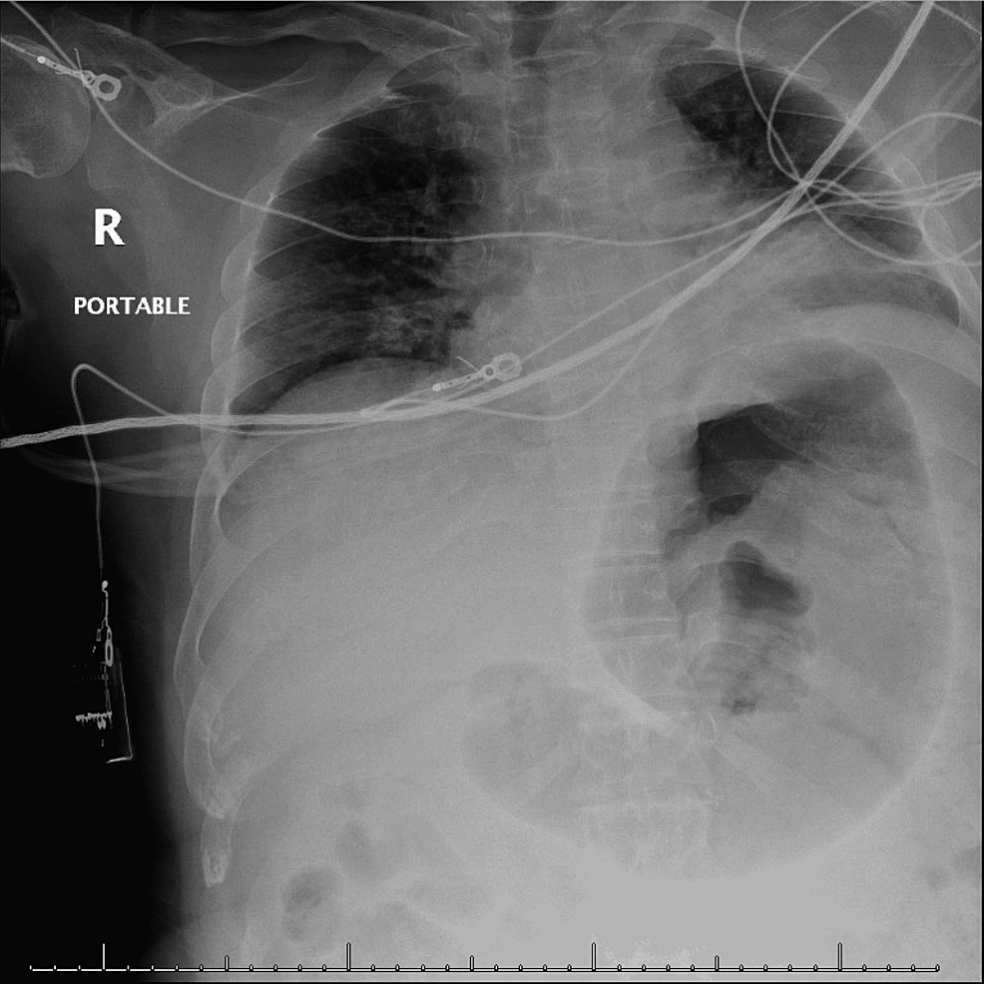
Chest x-ray Chest x-ray reveals poor inspiratory effort versus shallow respiratory volume with only six posterior ribs in the thoracic field. There is vascular crowding with questionable congestion and cardiomegaly.

Neurosurgical consultation recommended urgent hematoma evacuation. Preoperative history was unobtainable due to the patient's altered mental status. His mother provided limited collateral information that suggested a history of severe obstructive sleep apnea as a child with prior elective tracheostomy at eight years old and reversal at 11 years old. The patient was noted to be a difficult intubation in the past, but his mother could not specify on how the previously intubations were successfully achieved. Review of trauma bay chest x-ray (Figure 2) displayed shallow respiratory volume and vascular crowding without acute pathology.

Intraoperatively, the patient was positioned supine with an assistant providing manual in-line head and neck stabilization. Preoxygenation was achieved via a face mask with 100% inspired oxygen until the end-tidal oxygen concentration was sufficiently greater than 90%. Anesthetic induction was initiated with lidocaine 80 mg, propofol 100 mg, and rocuronium 60 mg. Sugammadex 16 mg/kg and a size 4 intubating laryngeal mask airway (LMA) were kept on hand in the event of an emergency. Rapid sequence intubation was attempted using video-assisted laryngoscopy with a MacIntosh 3 blade. Upon insertion into the oropharynx, the patient was noted to have precipitously desaturated to 60% on continuous pulse oximetry monitoring. The laryngoscope was immediately removed, oropharyngeal secretion was suctioned, and the LMA was inserted with adequate ventilation and oxygenation. At this point, the patient suddenly became bradycardic with a heart rate in the 40s. Intravenous epinephrine 0.5 mg was administered. However, the patient subsequently developed asystole. Advanced cardiac life support (ACLS) protocol was initiated with an additional 0.5 mg intravenous epinephrine given. Ventilation was continued via LMA and return of spontaneous circulation (ROSC) was achieved within one minute. A fiberoptic bronchoscope was introduced through the LMA with subsequent definitive airway establishment via a cuffed 7 mm endotracheal tube.

Neurosurgery decided to delay the surgery given the cardiopulmonary event and a repeat CT head was obtained to re-evaluate the need for urgent craniotomy. Fortunately, on the follow-up CT head, the subdural hematoma remained stable in size and neurosurgery opted for conservative medical management. The patient remained intubated and was transferred to ICU for close monitoring. Ultimately, he had an uncomplicated extubation the following day and was discharged home on hospitalization day six after return of normal neurological function without further surgical intervention.

## Discussion

The anesthetic care for patients with achondroplasia is complex, especially with limited literature on management in the acute setting. These cases are rarely encountered but have the potential for catastrophic outcomes if precautions are not taken in advance. In contrast to elective surgeries, thorough preoperative testing and objective surgical risk stratifications are unlikely available in an emergency. Our case, although not novel, attempts to address the possible problematic features of achondroplasia and highlight the anesthetic considerations in urgent situations where limited information is known.

In comparison to the average human anatomy, these patients are prone to have difficult airway features (e.g., depressed nasal bridge, maxillary hypoplasia, small oral orifice, macroglossia, adenotonsillar hypertrophy, shortened thyromental, tracheobronchomalacia) that could limit the range of motion in their neck, impede sufficient seal for face mask ventilation, and place them at increased risk for upper airway obstruction [3,4]. Structurally, these patients also have a narrower foramen magnum that is funnel-shaped and special care must be taken during intubation positioning and oropharyngeal manipulation [3,4]. Although statistically unreported in adults, one prospective study in achondroplastic infants reported radiological findings for craniovertebral stenosis and craniomedullary compression in 58% and 35% of their study subjects, respectively [5,6]. This emphasizes the accentuated risk for spinal compression and neurological issues with head and neck manipulation. As a result of these fragile cervical anomalies, anesthesia providers should avoid medications that induce myotonic fasciculations and refrain from rapid sequence induction with a depolarizing neuromuscular blocking agent (i.e., succinylcholine). Subsequently, these spasms can preclude ventilatory efforts, worsen neck hyperextension, and exacerbate atlantoaxial instability with the possibility for atlanto-occipital dislocation and cervicomedullary compression.

Patients with achondroplasia also present with thoracic and chest wall abnormalities, including reduced anteroposterior dimensions, rib hypoplasia, scoliosis, and pectus excavatum which can heavily diminish their lung volumes [7]. Other pulmonary comorbidities including central and obstructive sleep apnea, pulmonary hypertension, obesity, obstructive and restrictive lung diseases can synergistically deteriorate their respiratory capacities [4]. Perioperative administration of sedatives, such as benzodiazepines, and opioid analgesics should also be limited unless absolutely required due to their inhibition of spontaneous respiratory drive and further disrupt oxygen exchange. In Table 1, we summarized some the clinical features in achondroplasia and their complementary anesthetic concerns.

**Table 1:** Clinical features and associated comorbidity of achondroplasia and their anesthetic concerns This table displays an unexhausted list of clinical features in achondroplasia and their associated comorbidities along with possible anesthetic problems and considerations when managing such patients [8].

Anatomical Areas & Organ Systems of Interest	Clinical Features and Associated Comorbidities	Possible Anesthetic Problems
Limbs	Disproportionately rhizomelic dwarfism, leg bowing, limited elbow extension, hypermobile hips, and knees	Consideration for surgical positioning and avoidance of injuries to limbs during adjustments
Craniofacial & Neck	Hydrocephalus, frontal bossing, depressed nasal bridge, maxillary hypoplasia, macroglossia, oromotor hypotonia, short thyromental distant, limited neck extention, foramen magnum stenosis/craniocervical stenosis, occipitalization of C1 vertebra, kyphosis of C2-C3 vertebrae, adenotonsillar hypertrophy	Decreased nasopharyngeal passage with collapsible larynx precluding direct laryngoscopy and placement of oral/nasopharyngeal adjunct airways
		Manipulation of the neck can lead to atlantoaxial subluxation, cervical dislocation, and cervicomedullary compression
		Avoidance of depolarizing muscle relaxant and fasciculation inducing agents in causing or complication fractures. Spastic neck hyperextension can lead to atlanto-occipital dislocation.
Cardiorespiratory	Airway malacia, thoracic narrowing with decreased anteroposterior dimension, rib hypoplasia, thoracic kyphoscoliosis, pectus excavatum, central and obstructive sleep apnea, pulmonary hypertension, obesity, obstructive or restrictive lung disease	Possible complete airway obstruction from different airway malacias with difficulties in achieving adequate ventilation
		Impaired functional air spaces for optimal preoxygenation.
		Decreased respiratory volumes and oxygen reserves resulting in shorter time to desaturation
		Avoid preoperative sedatives whenever possible.
		Long-standing issues need further consideration for cardiac insufficiency and cor pulmonale.
Spine	Thoracic kyphoscoliosis, lumbar hyperlordosis, decreased distance between vertebral pedicles and accessible space, and spinal canal stenosis	Obstacles for neuraxial anesthesia (i.e., epidural, spinal) with possible hazardous spinal cord damage and neurological sequalae
		Ultrasound guidance can facilitate intrathecal placement.
		Dosing and volume of neuraxial anesthetic may be difficult to be determined due to spinal abnormalities and unpredictable spread with greater potential risk for high spinal.
		Epidural is preferred over spinal anesthesia with careful titration as needed.

As a result of the numerous difficult airway characteristics and rapid desaturation time associated with achondroplasia, a detailed preoperative history can shed light on previous successful approaches, provide key factors to avoid, and influence feasible choices in other anesthetic modalities (e.g., regional anesthesia, intravenous anesthesia). In emergency situations where preoperative history and patient chart review are unavailable, reliance on collateral information, even if limited, can be helpful. Providers should also note that while detailed history or collateral information can assist in management decisions, investigation should not delay an emergency live saving procedure.

Under circumstances where general anesthesia is necessary, awake fiberoptic intubation is indicated in alert and hemodynamically stable patients to secure an airway prior to induction of anesthesia [9]. Of note, an awake fiberoptic intubation was contraindicated in our patient due to his altered mental status and inability to follow commands. Some other contraindications to awake fiberoptic intubations includes patients that are unresponsive, having sustained orofacial traumas, oropharyngeal hemorrhage, basilar skull fractures, laryngeal traumas, allergies to local anesthesia, or concomitant coagulopathies [9]. In cases where awake fiberoptic intubation is contraindicated, an anesthesia contingency plan (e.g., video-assisted laryngoscopy with rescue supraglottic airways, elective surgical airways) can mediate and bypass the probable dilemmas anticipated in achondroplasia. Although uncommon, an ominously feared problem in these patients is the potential for complete airway obstruction occurring post-induction with a resultant cannot-intubate-cannot-ventilate scenario. In these predicaments, it is recommended to use alternative intubation approaches (e.g., alternative laryngoscope blade, intubating supraglottic airway, flexible bronchoscopy) with simultaneous preparation for emergent invasive airways (e.g., surgical cricothyroidotomy, needle cricothyroidotomy with pressure-regulated devices, large-bore cannula cricothyroidotomy, surgical tracheostomy). We also note that some anesthesia providers might not be confident in performing surgical airways in an emergency context with future plans for simulated training in these skills. In one study, 87% of the surveyed anesthesia providers at a tertiary hospital reported that they have not performed an emergent surgical airway before, and most of these providers do not feel confident in performing these procedures in a cannot-intubate-cannot-ventilate scenario [10]. In these instances, standby otolaryngologists or surgeons can assist with surgical airways in rapid succession when alternative intubation approaches have failed. Optionally, elective surgical airways (e.g., retrograde wire-guided intubation, percutaneous tracheostomy, surgical cricothyroidotomy) have gained increasing popularity within recent years. These procedures can be considered on an individualized basis as the initial step when severely complicated airways are anticipated [11]. Our institution follows the American Society of Anesthesiology emergency airway guideline, but this may vary from institution to institution [12]. Table 2 outlines our supplementary tactics in optimizing achondroplastic patients for endotracheal intubation with regard to limiting apneic durations and minimizing hypoxia and subsequent complications.

**Table 2:** Difficult airway and general anesthesia optimization in achondroplasia This table highlights some the preventative measures in anticipating achondroplastic difficult airways. These steps prioritize decreasing the apneic time given the low oxygen reserve in these patients and provide alternative options and advanced measures if an airway cannot be definitively established [13]. LMA: laryngeal mask airway; ET: endotracheal tube

Things to Consider in Anticipation of Achondroplastic Airways
Have difficult airway equipment cart in room (e.g., oral/nasopharyngeal adjuncts, SGA/LMA airways, intubating LMA, fiberoptic bronchoscope, bougies, cricothyroidotomy kit)
Avoid preoperative sedatives to retain spontaneous ventilation unless absolutely required
Prophylactic bronchodilators if available without contraindications
Positive airway pressure can assist in achieving adequate preoxygenation. In alert and oriented patients, an upright position can maximize oxygen saturation.
High-flow nasal canal for maximum apneic oxygenation
Awake intubation if not contraindicated (e.g., orofacial fractures, inability to follow commands, intolerable laryngospasms)
Consider elective surgical airway if anticipating severely complicated airways with comorbities
Continuously monitor oxygen saturation. Stop intubation process and bag mask ventilate patient when oxygen saturation falls below 90% based on clinicians’ judgment.
Adequate anesthetic induction dosages for achondroplastic patients In rapid sequence induction, it is advisable for equivalent dosages to an average-stature adult of that age rather than weight-based dosing with titration in elective cases)
Assistance with head and neck stabilization (e.g., manual in-line stabilization, cervical collar)
Use video-assisted laryngoscopy on first attempt. Limit to two attempts. Avoid fixation on failed techniques and try alternative methods.
Ensure variable range of endotracheal tubes sizes are available. Usually a smaller than age-estimated ET tube size is needed for smaller their airway anatomy
Surgery team in room in case of emergency invasive airway needed (e.g., cricothyroidotomy, large-bore cannula cricothyrotomy)
In emergency cardiac surgeries with perfusionists on standby, pre-cannulation for extracorporeal membrane oxygenation can prevent hypoxia, but should not delay the urgency of the operative procedure

Lastly, we would like to acknowledge other complex drawbacks and limitations in our case. Our patient’s expeditious desaturation with induction of anesthesia can presumably be from atelectasis leading to worsening ventilation-perfusion mismatch, and partly attributable to the significant reduction in residual lung volumes. We could have better optimized this by using prophylactic bronchodilators (e.g., albuterol) prior to induction and placement of high-flow nasal cannula for continuous apneic oxygenation. In conjunction with a sudden onset bradycardia that was unresponsive to epinephrine and further development of pulseless electrical activity, it is hard to exclude possible suspicion for direct cervicomedullary compression [14]. Fortunately, ACLS was promptly initiated with swift achievement of ROSC. Successful ventilation with an intubating LMA was crucial in oxygenating the patient and allowed for definitive endotracheal intubation via fiberoptic bronchoscopy. We chose to perform this alternative intubation method over a surgical airway procedure due to the readily available instruments in our operating room as well as having a less traumatic airway outcome. If this had failed, however, an emergent surgical airway would have been implemented. As exampled by this case, a multitude of complexities can arise when attending to achondroplastic patients in the urgent traumatic setting. Furthermore, limited encounters with these patients hinders active training experience, especially if unfamiliarized physicians stumble upon these cases for the first time. This article aims to shed light on the anesthetic concerns that physicians should integrate into the management of achondroplastic dwarfs.

## Conclusions

Achondroplasia, although rare, is likely to be encountered at least once in a medical provider’s career. These patients often present with anatomical and physiological alterations that can impede the safety of different anesthetic modalities, of which a thorough evaluation of the patient and available options should be considered on an individualized basis. If these patients require emergency operations under general anesthesia, difficulty securing an airway is among one of the most prominent issues, especially if the patient is unable to follow commands and preoperative history is unknown or limited. Our case emphasizes awareness in achondroplastic airway dilemmas and perioperative considerations in preventing some of the commonly foreshadowed challenges in their anesthetic management.
